# Impact of Cancer Stem Cells on Therapy Resistance in Gastric Cancer

**DOI:** 10.3390/cancers14061457

**Published:** 2022-03-11

**Authors:** Maddalen Otaegi-Ugartemendia, Ander Matheu, Estefania Carrasco-Garcia

**Affiliations:** 1 Cellular Oncology Group, Biodonostia Health Research Institute, 20014 San Sebastian, Spain; maddalen.otaegi@biodonostia.org (M.O.-U.); ander.matheu@biodonostia.org (A.M.); 2 CIBER de Fragilidad y Envejecimiento Saludable (CIBERfes), 28029 Madrid, Spain; 3 IKERBASQUE, Basque Foundation for Science, 48009 Bilbao, Spain

**Keywords:** gastric cancer, gastric cancer stem cells, therapy resistance

## Abstract

**Simple Summary:**

Therapy resistance represents one of the major obstacles to curing cancer. Particular populations of tumor cells, known as cancer stem cells, are responsible for this resistance and, therefore, constitute key targets in the disease. In this review, we address the impact of cancer stem cells on therapy resistance in gastric cancer and we highlight the relevance of the different regulators of these cells that have been linked to resistance.

**Abstract:**

Gastric cancer (GC) is the fourth leading cause of cancer death worldwide, with an average 5-year survival rate of 32%, being of 6% for patients presenting distant metastasis. Despite the advances made in the treatment of GC, chemoresistance phenomena arise and promote recurrence, dissemination and dismal prognosis. In this context, gastric cancer stem cells (gCSCs), a small subset of cancer cells that exhibit unique characteristics, are decisive in therapy failure. gCSCs develop different protective mechanisms, such as the maintenance in a quiescent state as well as enhanced detoxification procedures and drug efflux activity, that make them insusceptible to current treatments. This, together with their self-renewal capacity and differentiation ability, represents major obstacles for the eradication of this disease. Different gCSC regulators have been described and used to isolate and characterize these cell populations. However, at the moment, no therapeutic strategy has achieved the effective targeting of gCSCs. This review will focus on the properties of cancer stem cells in the context of therapy resistance and will summarize current knowledge regarding the impact of the gCSC regulators that have been associated with GC chemoradioresistance.

## 1. Gastric Cancer

Gastric cancer (GC) is a global health problem that accounts for more than 1 million new cancer cases annually and that constitutes the fourth leading cause of cancer death worldwide (1,089,103 new cases and 768,793 deaths in 2020) [1]. The average 5-year survival rate for GC patients is 32%, being of 6% for patients presenting with cancer spread to distant parts of the body. The prognosis for patients with recurrent disease or metastasis is dismal, with a median survival of only 8 months [2].

GC is a multifactorial disease that is linked to both host and environmental factors. The major risk factor for GC development is chronic infection by *Helicobacter pylori* (*H. pylori*) [3], a Gram-negative microaerophilic bacterium that colonizes the gastric mucosa and induces a series of sequential alterations that begin with non-atrophic gastritis, which eventually progresses to multifocal atrophic gastritis, intestinal metaplasia and dysplasia [4]. Other factors that are associated with an increased risk of GC are smoking, alcohol consumption, a high-salt diet, a sedentary lifestyle, obesity, or gastroesophageal reflux disease [5].

### 1.1. Classification

Most gastric cancers (around 90%) are adenocarcinomas and arise from the glands of the gastric mucosa. At the histological level, different types of gastric tumors can be distinguished. The Lauren’s classification, which dates from 1965, classifies GC into intestinal (50%), diffuse (33%) and mixed (17%) types [6]. The diffuse type presents cancer cells spreading through the stroma, is predominant among younger patients and exhibits poor differentiation and a worse prognosis than intestinal type cancers. The intestinal type is predominant in males and is more strongly associated with *H. pylori*, chronic atrophic gastritis and intestinal metaplasia [7]. The histological classification established by the World Health Organization (WHO) in 2010 defines four GC subtypes: tubular, papillary, mucinous and poorly cohesive (including signet ring cell carcinoma) [8]. Tubular tumors are composed of glandular structures and are the most frequent. The papillary subtype is more common in older patients and is usually associated with spread to the lymph nodes and the liver. Mucinous gastric carcinoma is atypical and is characterized by the presence of abundant extracellular mucin (≥50% of tumor volume). Poorly cohesive carcinomas are diffuse and generally composed of signet ring cells and diverse cancer cells that resemble histiocytes, lymphocytes or plasma cells.

Although histological classifications exhibit certain prognostic connotations, they have very limited utility in the clinical management of GC due to the molecular heterogeneity of the disease.

At the molecular level, high-throughput technologies have elucidated the molecular landscape of GC. As a result, two main molecular classifications of GC have been proposed. The Cancer Genome Atlas (TCGA) network proposed a classification of GC into Epstein–Barr virus (EBV)-positive (EBV+), microsatellite-unstable (MSI), genomically stable (GS) and GC presenting chromosomal instability (CIN) [9]. The EBV+ group is characterized by EBV positivity, is more frequent in males (81%), and exhibits DNA hypermethylation (not affecting *MLH1*), *PIK3CA*-activating mutations (in around 70% of tumors) and the amplification of *JAK2*, *PDL1* and *PDL2*. MSI tumors are more common at older ages (median age of 72 years) and are characterized by high mutation rates and *MLH1* hypermethylation. GS tumors are more frequent in younger individuals (median age of 59 years), are mainly diffuse and are enriched in mutations that affect *RHOA* and *CDH1*. Finally, CIN tumors present focal amplification of receptor tyrosine kinases, a high frequency of *TP53* mutations (73%), and amplification of cell cycle-related genes [9]. For its part, the Asian Cancer Research Group (ACRG) defined a molecular classification that exhibits prognostic significance. This classification distinguishes the following subtypes of GC: GCs showing high MSI, microsatellite-stable GCs with active TP53 (MSS/TP53+), microsatellite-stable GCs with inactive TP53 (MSS/TP53-) and microsatellite-stable tumors expressing an epithelial–mesenchymal transition (EMT) signature (MSS/EMT) [10]. MSI cases, generally intestinal, are diagnosed at earlier stages and present the best prognosis (mean survival of 77.8 months). This subtype is associated with *MLH1* loss, *ARID1A*, *KRAS* and *ALK* mutations, and mutations in genes belonging to the PI3K-mTOR pathway. The MSS/TP53- subtype (mean survival of 59.8 months) is mainly intestinal and has the highest rate of *TP53* mutation, being also common in this subtype the amplification of *ERBB2 (HER2)*, *CCNE1*, *MYC* and *EGFR*. The MSS/TP53+ group has the second-best prognosis (mean survival of 66.9 months) and agglutinates most EBV+ cases. It occurs more frequently in males (72.2%) and is characterized by *APC*, *ARID1A*, *KRAS*, *PIK3CA* and *SMAD4* mutations. For its part, the MSS/EMT subtype is diagnosed in younger patients (median age of 53 years) and presents the worst outcome (mean survival of 42.6 months), with a higher probability of peritoneal seeding. These tumors are mostly diffuse (80.4%), exhibit low mutation rates, and present a gene expression pattern that is related to EMT.

### 1.2. Treatment

The treatment for GC includes surgical resection (feasible in about 20% of cases), chemoradiotherapy and targeted therapy. There is no single regimen that has been established for the chemotherapeutic treatment of GC, existing different available drugs that are administered as single agents or in combination. These agents include platinum derivatives (cisplatin or oxaliplatin), pyrimidine analogs (5-fluorouracil (5-FU)), anthracyclines (doxorubicin, epirubicin), taxanes (paclitaxel or docetaxel) or the camptothecin derivative irinotecan. Operable GC undergoes surgery (total or partial gastrectomy plus lymphadenectomy) and is generally treated with neoadjuvant chemotherapy and adjuvant chemoradiotherapy. Advanced GC is treated in first-line with pyrimidine- and platinum-based compounds, in combination with the antibody Trastuzumab against Epidermal Growth Factor Receptor 2 (HER2) in HER2-positive cases. Second-line treatment consists of paclitaxel and the anti-angiogenic antibody Ramucirumab, which recognizes Vascular Endothelial Growth Factor Receptor 2 (VEGFR2). Finally, the third-line option consists of chemotherapy with irinotecan. Regarding immunotherapy, Pembrolizumab, a monoclonal antibody against the programmed death receptor 1 (PD-1), is administered in chemotherapy refractory disease cases that are positive for PD-L1 or that exhibit high MSI [11]. More recently, in April of 2021, and based on the results of the CHECKMATE-649 trial (NCT02872116) [12], the antibody Nivolumab, which also targets PD-1, has been approved in combination with pyrimidine- and platinum-based chemotherapy for the treatment of advanced GC.

Unfortunately, despite the advances that have been made in chemotherapy and the implementation of targeted therapies, the majority of patients still experience disease progression after receiving these treatments. In this sense, none of the therapeutic agents that are currently available is able to target cancer stem cells (CSCs), and this is a highly decisive aspect in the failure of therapies.

## 2. Cancer Stem Cells

### 2.1. Origin

**Figure 1:**
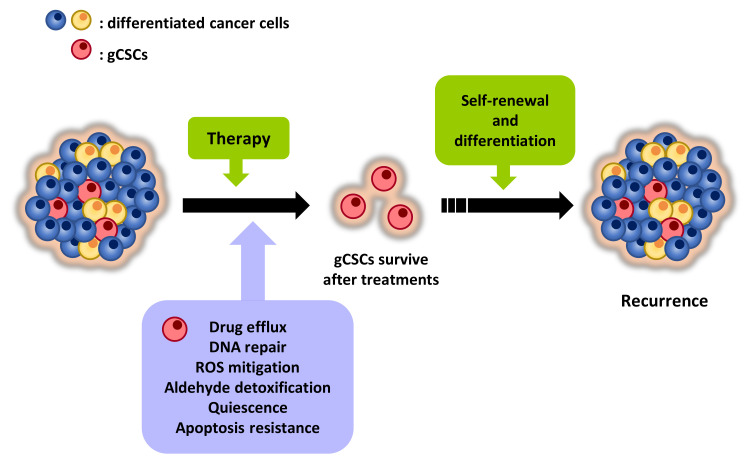
gCSCs are responsible for therapy failure and relapse. gCSCs represent a minority subpopulation of cancer cells within the tumor bulk that are characterized by unique properties that make them resistant to conventional therapies. As a consequence, they promote tumor recurrence and, with it, dismal prognosis.

Tumors are highly heterogeneous entities that are composed of different populations of cancer cells and other types of cells, such as endothelial, infiltrating immune or stromal cells. Among cancer cells, a small proportion exhibiting properties of stem cells (SCs) represent the population of CSCs (Figure 1). CSCs were first isolated from acute myeloid leukemia (AML) in 1997 [13], being in the successive years isolated from multiple solid tumors, such as breast, brain, colon or GC [14,15,16,17]. CSCs exhibit an indefinite self-renewal capacity and can differentiate originating phenotypically diverse tumor cells. These characteristics allow their long-term repopulation for tumor maintenance and establish CSCs as the major source of heterogeneity in tumors. In this regard, a growing body of evidence supports the notion that tumors are organized in a hierarchical fashion that is governed by CSCs, which are considered to be responsible for tumor origin, therapy resistance, recurrence and metastasis [18,19,20,21].

Regarding their origin, it has been proposed that CSCs might arise from the transformation of the populations of SCs resident in adult tissues, which sustain life-long tissue homeostasis. In the stomach, the epithelium is in continuous renewal fueled by the population of gastric stem cells (gSCs) [22]. Different subsets of potential gSCs have been identified in the gastric glands of the different anatomical areas of the stomach through the development of in vivo cell lineage tracing studies in mice. For instance, Lgr5+ cells placed at the base of the pyloric glands have been shown to act as multipotent SCs in the renewal of gastric units [23]. Likewise, the Sox2+ cells that are located in the glands of the pylorus and corpus can self-renew and are able to originate the mature cell types of the gastric glands [24]. Moreover, in contexts of injury, the Lgr5+ cells in the corpus glands and the Villin+ cells in the isthmus of the antral glands have been shown to participate in epithelial renewal [25,26]. In an attempt to identify the cellular origin of GC, different studies have introduced transforming alterations in specific stem cell compartments in mice. In this setting, both the deletion of the tumor suppressor *Adenomatous polyposis coli* (*Apc*) in Lgr5+ cells and the deletion of the GC suppressor *Kruppel-like factor 4* (*Klf4*) in Villin+ cells resulted in the appearance of gastric adenomas [23,27], reflecting that multiple stem/progenitor pools might be susceptible to oncogenic transformation.

The GC inductor *H. pylori* has been implicated in the transformation of the resident gSCs. Thereby, studies in GC patients demonstrated that *H. pylori* reaches the gastric glands and affects the genomic integrity of the population of LGR5+ SCs, which is expanded in patients affected by the bacterium [28,29,30]. Additionally, regarding *H. pylori*’s involvement in the appearance of gCSCs, it has been proposed that this population could derive from bone marrow-derived gastric SCs (BMDSCs) that have been recruited to the stomach in response to *Helicobacter* infection [31,32].

### 2.2. CSCs: Characteristics and Therapy Failure

The particular properties of CSCs are closely related to therapy resistance and tumor progression (Figure 1). In this sense, a critical feature of CSCs is their ability to self-renew and differentiate giving rise to heterogeneous tumor cells. As a consequence, CSCs sustain tumors and are responsible for tumor heterogeneity, an aspect that foments resistance phenomena and that compromises the effectiveness of therapies. According to this capability, in the experimental setting, CSCs are able to recapitulate the primary tumors from which they proceed when they are serially transplanted into immunocompromised mice. Moreover, when cultured in non-adherent conditions in serum-free medium supplemented with growth factors, CSCs grow forming spheroid structures (known as oncospheres, tumorspheres, etc.) and display self-renewal potential, whilst non-CSCs demise [33,34].

**Figure 2:**
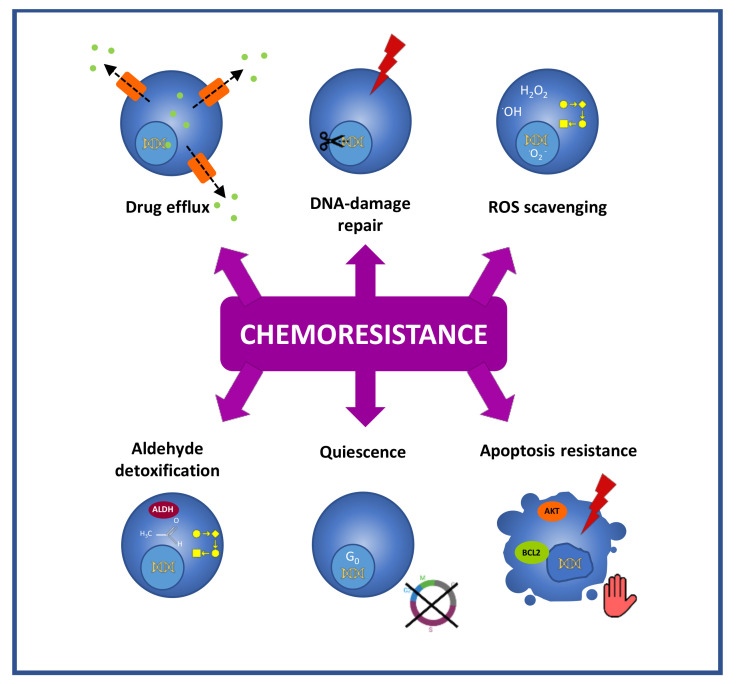
Chemoresistance mechanisms exhibited by gCSCs. gCSCs evade the effects of treatments through different mechanisms, such as active drug efflux, high DNA repair activity, efficient ROS scavenging, the high detoxification of aldehydes, their quiescence status, and apoptosis resistance mechanisms.

Another distinctive feature of CSCs that is linked to chemoresistance is their high drug efflux capacity due to the expression of ATP-binding cassette membrane transporters [35,36,37] (Figure 2). These transporters are located at the plasma membrane and actively efflux from cells functionally and structurally unrelated hydrophobic and hydrophilic compounds, including chemotherapeutic agents such as taxanes, anthracyclines, camptothecins or vinca alkaloids [38]. This action results in decreased intracellular drug accumulation, allowing the CSCs to evade the effect of treatments. Thus, based on their low intracellular accumulation of fluorescent dyes such as Hoechst 3342, CSCs have been designated as the side population (SP) in flow cytometric analysis [35]. Nonetheless, this approach is controversial since different works have described SP populations that do not exhibit properties of CSCs [39,40,41]. Furthermore, the cytotoxic character of Hoescht 3342 is a matter of debate and some authors state that the phenotypic differences between the cells that do and do not belong to the SP fraction may be influenced by the toxic effect of the dye itself [42]. In the case of GC, the analysis of SP populations isolated from GC cell lines has provided inconsistent and cell line-dependent results [39,40,43,44].

Like ionizing radiations, different anti-cancer agents cause DNA damage. Cisplatin and oxaliplatin induce covalent crosslinks between DNA bases, irinotecan produces DNA single-strand breaks, cyclophosphamide and temozolomide alkylate DNA, etc. [45]. In this context and representing an important obstacle for the efficacy of genotoxic therapies, CSCs exert an enhanced DNA repair activity due to the overexpression of DNA repair machinery components such as the endonuclease ERCC1, the replicase RIF1, or the ubiquitin-conjugating enzyme UBE2 [45] (Figure 2).

CSCs also display an enhanced efficiency in the mitigation of reactive oxygen species (ROS), a faculty that protects this population against ROS-mediated damage induced by chemo- and radiotherapy [46] (Figure 2). Notably, in GC it has been shown that gCSCs present high levels of reduced glutathione (GSH), the major intracellular antioxidant [47], and this feature has been associated with enhanced resistance to cisplatin, docetaxel [47] and 5-FU [48].

Moreover, in relation to DNA protection, CSCs exhibit high aldehyde dehydrogenase (ALDH) (All abbreviations are listed in glossary (Table S1)) activity (Figure 2). This characteristic enables the efficient detoxification of aldehydes, which induce DNA damage [49], and is also exploited for the isolation of CSCs based on the detection of an ALDH’s reaction fluorescent product (ALDEFLUOR™ assay) [50]. Besides, ALDH contributes to resistance through ROS scavenging [51] and through the metabolism of cyclophosphamide [52].

Another challenge in CSCs targeting is the fact that they can remain in a quiescent state (Figure 2). This aspect enables the evasion of most of the anticancer drugs, which target processes that are linked to proliferative states, such as DNA synthesis or mitosis. Thus, 5-FU interferes with DNA synthesis [53], and anthracyclines insert between adjacent DNA base pairs and block DNA and RNA synthesis [54], while paclitaxel and its semisynthetic analogue docetaxel are microtubule-stabilizing agents that prevent mitosis [55]. Quiescence allows the identification and isolation of CSCs through labeling procedures in which CSCs, as non-proliferative cells, retain intracellular dyes over time, which, in contrast, are diluted in the progeny in the case of proliferative cancer cells. Notably, quiescence is a reversible state, and, after therapy, these quiescent CSCs have the capacity to re-enter the cell cycle and repopulate tumors [56]. In GC, in vitro results obtained in cell models stated that PLK1 and RSK1 are relevant players in the switch of gCSCs between proliferative and quiescent states [57].

They are also relevant in chemoresistance, the mechanisms that are available in CSCs to avoid apoptosis after treatments (Figure 2). CSCs develop apoptosis evasion through different mechanisms that involve the overexpression of antiapoptotic proteins (Bcl-2 family, IAPs) [58], the overactivation of survival signaling through the PI3K/AKT pathway [59], the downregulation of death receptors [60,61], or the upregulation of the anti-apoptotic protein c-FLIP [62], which blocks caspase activation.

## 3. Regulators of gCSCs Linked to Therapy Response

Being CSCs critical players in cancer therapy resistance, different gCSC regulators have been related to the low response to radio/chemotherapy in GC cell models in vitro, xenograft models in vivo and GC patients. Next, we review the current knowledge regarding the critical regulators of gCSCs that have been linked to therapy response (Table 1).

### 3.1. LGR5

The human leucine-rich repeat-containing G-protein coupled receptor 5 (LGR5) is a member of the superfamily of G protein-coupled transmembrane receptors (GPCRs). LGR5 enhances WNT signaling through its activity as a receptor for R-spondins, which act as agonists of the pathway and synergize with the WNT proteins [63]. Moreover, LGR5 itself is a target gene of the WNT pathway [64]. As we have indicated above, different studies indicate that LGR5 is a marker of the homeostatic gSCs [23] that are susceptible to oncogenic transformation [28,29,30]. LGR5 has been linked to outcome and therapy resistance in GC. In vitro results have shown high LGR5 expression in GC cell line-derived spheres, as well as enhanced sphere growth, migration and resistance to oxaliplatin in GC cells after ectopic LGR5 overexpression [65]. Additional works performing LGR5 modulation in GC cell lines revealed its involvement in resistance to oxaliplatin and 5-FU [66,67]. In GC patients, the high LGR5 expression in tumor tissue is associated with adverse clinicopathological features and a dismal prognosis [66,68,69,70]. Interestingly, LGR5 has been linked to therapy response in GC patients. The research performed by Bauer and collaborators including GC patients treated with neoadjuvant chemotherapy based on platinum compounds or 5-FU, explored *LGR5* expression in pre-therapeutic biopsies and resected tumors, finding increased expression of this marker in residual tumor cells in post-therapeutic samples [71]. Furthermore, two independent studies reported that in GC patients treated with neoadjuvant chemotherapy, the rate of positive LGR5 expression was higher in the resected specimens exhibiting poor tumor regression compared to regressed tumors [66,72]. These results are compatible with a potential enrichment in drug-resistant tumor cells expressing LGR5 after chemotherapy and, altogether with the in vitro findings, highlight the significance of this gCSC marker in therapy response.

### 3.2. CD44

CD44 is a widely expressed cell surface adhesion molecule that binds to components of the extracellular matrix (ECM), mainly hyaluronic acid (HA), and other non-ECM ligands such as interferon-α or serglycin. CD44 is involved in a broad plethora of cellular processes, including cell–cell and cell–ECM interactions, signal transduction or lymphocyte activation [73]. The genomic structure of *CD44* contains a variable region that comprises 10 exons that are subject to alternative splicing. As a result, a broad diversity of CD44 isoforms is possible. The standard form (CD44s) lacks the variable region and is ubiquitously expressed, while the expression of the different variants (CD44v) is more restricted to specific contexts, including cancer [74,75,76].

CD44 regulates adult and embryonic hematopoietic stem cells [77,78]. In the stomach, CD44 expression is restricted to the base of the gastric crypts, the location where the stem and progenitor cells reside [79]. CD44 has been also identified as a CSC marker/regulator in many types of cancers, such as in breast [14], colorectal [80,81] and non-small-cell lung cancer (NSCLC) [82], among others. In GC, CD44 was the first CSC biomarker proposed when Takaishi and collaborators found that the fraction of CD44+ cells isolated from GC cell lines exhibited self-renewal capacity in vitro and tumorigenic potential in immunodeficient mice, being these properties abrogated by *CD44* silencing. Notably, these CD44+ cells exhibited increased resistance to radiation, 5-FU and etoposide [17]. As expected, GC sphere cultures express high levels of CD44 [83] and *CD44* knockdown in GC cells confers chemosensitivity [84]. Similar studies showed that the CD44+ cells derived from GC cell lines were able to form highly self-renewing spheres, exhibited an increased capacity for migration, invasion and anchorage-independent growth, and were resistant to 5-FU, cisplatin, paclitaxel and radiation [57,85]. In accordance, GC cells that are resistant to 5-FU treatments, which exhibit enhanced CSC properties in vitro and tumorigenicity in vivo, were enriched in CD44 expression [86]. Similar findings have been obtained in GC tissue-derived 3D organoids that were resistant to 5-FU as a result of selective pressure with increasing concentrations of the drug [87]. Moreover, the frequency of CD44+ cells increases in 2D and 3D GC cultures after acute treatments with docetaxel [88]. Of note, circulating tumor cells (CTCs) expressing CD44 isolated from patients have been shown to exhibit chemoresistance and multipotency ex vivo [89].

Multiple studies have linked CD44 to GC outcome and critical aspects of the disease that are related to gCSCs. Thus, the analysis of gastric tissue samples from patients suffering gastric dysplasia and early GC suggests that the emergence of gCSCs triggered by *H. pylori* infection may involve the induction of CD44 [90]. Notably, the high CD44 expression in GC is associated with a larger tumor size [91,92], a lower grade of differentiation [92,93,94], tumor relapse [94,95], lymph node invasion [91,96], metastasis [91,96,97] and reduced survival [84,85,91,92,93,94,95,98]. Besides, the presence of CD44+ cancer cells at the invasive front of gastric tumors is associated with poor prognosis [99], and the presence of circulating CD44+ tumor cells in patients correlates with tumor stage and depth, vascular invasion, metastasis and reduced survival [100,101]. Regarding therapy response in patients, high CD44 expression has been associated with disease progression in GC patients treated with FOLFOX (5-FU, oxaliplatin and leucovorin) [85]. In addition, the results of Wang et al. showed reduced expression of CD44 in post-therapeutic samples of GC patients exhibiting response to combined docetaxel, cisplatin and capecitabine. They also observed higher CD44 expression in preoperative biopsies from responsive patients compared to those from non-responsive patients [93]. These results could imply that CD44+ cells are susceptible to this combined treatment, but they must be taken with great caution due to the extremely limited number of patients included in the study (four responsive and four non-responsive).

Different studies have pointed out the relevance of CD44 variants in GC. For instance, the number of different CD44 variants expressed in tumors is related to prognosis, suggesting that these isoforms may carry out different activities that contribute to malignancy [99]. In 2002, the CD44v6 variant was associated with poor prognosis in differentiated GC [102]. More recently, a study has revealed that the exon-v6 specific removal from CD44v isoforms increases cell sensitivity to cisplatin and impairs the ability of GC cells to self-renew [103]. For its part, the study performed by Lau et al., which distinguished between the expression of CD44s and the expression of the variants CD44v6 and CD44v8-10, showed that CD44v8-10 is the predominant variant in GC xenografts. Moreover, they found that this variant was overexpressed in GC tissue with respect to normal gastric adjacent tissue, while the isoforms CD44s and CD44v6 were not overexpressed. The authors also determined through loss and gain-of-function procedures that the tumor-initiating potential of GC cells was attributable to the variant CD44v8-10 [104]. In accordance with this work, another study also found that the CD44v8-10 variant is the predominant in GC tissue [105]. It has also been shown that variant 9 (CD44v9) is relevant in GC. Thus, CD44v9 expression has been associated with reduced recurrence-free survival in GC patients treated with surgery and adjuvant tegafur (precursor of 5-FU) [106]. Moreover, these researchers found increased levels of GSH in CD44v9-positive tumors, linking this variant to an enhanced efficiency in the mitigation of ROS, a characteristic of CSCs linked to therapy failure.

The findings obtained from the study of specific CD44 variants allow us to glimpse the complexity of CD44 as a regulator of gCSCs in relation to therapy and point to the need for further research.

### 3.3. CD133

CD133 (or prominin-1) is a transmembrane cholesterol-binding glycoprotein that is located in plasma–membrane protrusions such as epithelial microvilli [107]. CD133 was first identified in human hematopoietic stem cells [108] and was then recognized as a SC marker in different tissues, including the pancreas, kidney or liver [108,109,110]. In the stomach, CD133+ cells, like CD44+ cells, have been identified in the stem cell zone of human gastric glands [79]. CD133 is a CSC marker in multiple types of cancer such as lung, pancreatic and ovarian cancer, as well as in melanoma [111,112,113,114]. As far as GC is concerned, CD133 is highly expressed in GC sphere cultures [83,84] and its silencing in GC cell lines impairs invasion, sphere formation and tumor growth [115]. In clinical samples, several studies have found an overexpression of CD133 in GC tissue with respect to the non-neoplastic gastric mucosa, as well as an association of this marker with adverse clinicopathological parameters such as tumor size, lymphatic/vascular infiltration, TNM stage, depth of invasion, distant metastasis and reduced survival [94,98,116,117,118,119]. Interestingly, the abundance of circulating CD133+ cells is also associated with lymphatic and venous invasion, and reduced survival of GC patients [120]. Regarding therapy response, different findings have linked CD133 expression to resistance. As such, CD133+ GC cells are more resistant to 5-FU than CD133- cells in vitro [121], and CD133 silencing enhances the sensitivity of GC cells to 5-FU [115,121] and cisplatin [122]. Furthermore, GC cells exhibiting acquired resistance to cisplatin overexpress CD133 [122]. In GC patients, a higher expression of CD133 in tumors before chemoradiation has been associated with tumor invasion depth, the presence of metastasis in distant organs, advanced TNM stage and reduced survival [94]. Similarly, the study of Hashimoto et al. associated high CD133 expression with worse prognosis in patients treated with adjuvant chemotherapy [118]. In line with this, more recently, a study performed with patients presenting stage III tumors revealed the association of high CD133 expression with shorter disease-free survival after post-surgical combined chemotherapy with platinum compounds and 5-FU in the presence or absence of docetaxel [119]. Therefore, targeting CD133+ cells in GC represents a worthwhile strategy to overcome chemoresistance.

### 3.4. HMMR

The hyaluronan mediated motility receptor (HMMR), also known as CD168, is a cell surface HA receptor (as CD44) that interacts with the HA of the extracellular matrix (ECM) through its carboxy-terminal region [123]. In addition, HMMR exhibits intracellular location and functions. HMMR binds to microtubules [124] and it is important for the stabilization of the mitotic spindle in human cells [125]. HMMR is highly expressed in different types of cancer, including in GC [126,127,128,129,130,131], in which it represents the most up-regulated gene in tumor tissue with respect to normal gastric tissue (data from the TCGA cohort) [132]. It has been shown that HMMR is critical for the maintenance of human embryonic stem cells [133] and also plays a role in the regulation of CSCs in breast cancer and glioblastoma [134,135]. In line with this, Zhang and collaborators found that the expression of HMMR is upregulated in gastric oncospheres and in GC cell lines resistant to 5-FU, being its silencing detrimental for the oncospheres and sensitizing GC cell lines and derived xenografts to the drug. Notably, high HMMR expression has been associated with poor outcome in GC patients treated with 5-FU, founding and association with lymph node dissemination, tumor relapse and reduced survival [131,132]. The current knowledge regarding HMMR in GC is very limited, yet it could represent a suitable target to impair gCSCs and chemoresistance.

### 3.5. E2F1

The transcriptional regulator E2F1 plays a well-known function in the control of cell cycle progression during the late G1/S phase, when it activates the transcription of genes that are required for DNA synthesis, S-phase entry and mitosis [136]. Additionally, E2F1 participates in the regulation of other cellular events such as apoptosis [137], senescence [138], DNA damage [139] and metabolism [140]. Members of the E2F family, including E2F1, have been shown to play a role in controlling stem and progenitor cell fate decisions and self-renewal [141]. In gastric glands, a study associated the specific gene expression profile in the oxyntic proliferative isthmus zone, the region that contains the stem/progenitor populations in the oxyntic epithelium of the stomach, with E2F1, indicating a role for this transcription factor in the regulation of gSCs [142]. E2F1 expression has been associated with poor prognosis in different types of cancers, such as in colorectal carcinoma (CRC) [143] or breast cancer [144]. In GC, a work published in 2008 found enhanced sensitivity to 5-FU in GC cell lines with ectopic upregulation of E2F1 and associated E2F1 immunopositivity to better prognosis in a cohort of GC patients receiving adjuvant 5-FU [145]. In contrast, more recently, different findings have linked E2F1 to CSCs, chemoresistance and poor prognosis. In vitro, enriched E2F1 expression has been shown in gastric oncospheres as well as enhanced oncosphere formation, migration and invasion after ectopic E2F1 overexpression, while E2F1 silencing produced the opposite effects [146]. In addition, E2F1 is upregulated in GC cell lines that are resistant to paclitaxel and cisplatin [147], whereas E2F1 silencing enhances the sensitivity of GC cell lines to cisplatin, doxorubicin, 5-FU, and paclitaxel plus cisplatin [146,147,148]. Of note, *E2F1* is overexpressed in GC tissue [142,146,147], wherein its expression is correlated with the expression of CSC markers [146], and predicts poor prognosis in patients treated with 5-FU [146,149].

### 3.6. ALDH

ALDHs constitute a family of detoxifying enzymes (19 members in humans) that catalyze the oxidation of intracellular active aldehydes and protect cells against oxidative stress [150]. Moreover, ALDHs are required for the biosynthesis of retinoic acid (RA), which participates in the regulation of different developmental processes, like the differentiation of neural or lymphohematopoietic stem cells [151,152]. ALDHs, particularly ALDH1A1 and ALDH1A3, are crucial in SCs [153], being its protective activity probably related to the longevity of this population. In fact, this high ALDH activity (measured by the Aldefluor™ assay) has been taken advantage of to isolate SC subpopulations from a variety of tissues, including brain, muscle, breast, prostate and liver [50]. In these tissues, cells presenting high ALDH activity (known as ALDH-bright) represent populations of self-renewing cells endowed with multilineage differentiation potential [50]. In the stomach, there is also evidence linking ALDH with gSCs, with the strongly ALDH1-positive cells being located specifically at the base of the crypts in the human stomach [154]. Besides, high ALDH activity is a feature for CSCs in many types of cancers [155]. In GC, the ALDH+CD44+/CD166+ signature has been proposed as the most tumorigenic phenotype among the cells derived from human primary gastric tumors [156]. Notably, cells presenting high ALDH activity isolated from GC cell lines are resistant to 5-FU and doxorubicin [156]. According to that, a recent work using primary cultures established from gastric tumors shows that residual cancer cells that are resistant to treatments with 5-FU, SN38 (analog of camptothecin), cisplatin and paclitaxel are enriched in ALDH1A3 expression [157]. In GC samples, Levi and collaborators observed that the number of ALDH1+ cells was low in normal human gastric mucosa and progressively increased in gastritis samples that were positive for *H. pylori*, gastritis samples positive for *H. pylori* with intestinal metaplasia, and gastric adenocarcinomas [158]. In GC patients, high ALDH1 expression has been associated with advanced TNM stage, depth of invasion, lymph node metastasis and reduced survival [159,160]. Moreover, another work revealed the association of high ALDH1A3 and ALDH1L1 expression with a worse prognosis in patients treated with 5-FU [161]. Altogether, these studies demonstrate the relevance of ALDH in therapy resistance prompted by gCSCs and encourage the design of specific therapies against ALDH.

**Table 1:** gCSC regulators that have been associated with therapy resistance in GC.

Marker	Preclinical Setting		Clinical Setting		
	CSCs In Vitro	Chemoresistance	Overexpression in GC	Association with Reduced Survival	Association with Poor Treatment Response
LGR5	[65]	[65,66,67]	[68,69]	[66,68,70]	[71,72]
CD44	[17,57,83,85,97]	[17,57,84,85,86,87,88,89,103]	[90,91,92,93,94,104,105]	[84,85,91,92,93,94,95,98,99,101,102,106]	[85,106]
CD133	[83,84,115]	[84,115,121,122]	[94,116,117,119]	[84,94,98,116,117,118,119,120]	[94,118,119]
HMMR	[132]	[132]	[131,132]	[131]	[131]
E2F1	[146]	[146,147,148]	[142,146,147]	[146,149]	[146,149]
ALDH	[156]	[156,157]	[158]	[160,161]	[161]

## 4. Different Approaches and Concerns in the Targeting of gCSCs

Currently, targeting gCSCs for the treatment of GC is still a challenge. However, in recent years, some clinical trials have tackled the analysis of gCSC regulators as biomarkers in the disease or have evaluated strategies aimed at targeting/inhibiting the regulators of gCSCs. In the case of CD44, the NCT01358903 trial analyzed the safety and antitumoral activity of the humanized monoclonal antibody RG7356 (also known as RO5429083) designed against CD44 in cancer patients harboring CD44-expressing solid tumors who experienced disease progression on standard therapy. In this trial, although RG7356 was well tolerated, its clinical efficacy was modest, producing disease stabilization in 21% of patients [162]. Remarkably, the NCT04427449 trial is evaluating the feasibility, safety and efficacy of a Chimeric Antigen Receptor (CAR) T-cell therapy (CAR-T) directed against CD44v6 in different cancers, including GC. Using a different approach, there is another trial focusing on the inhibition of this variant in advanced cancers, in this case through the usage of a compound (AMC303) exhibiting high affinity and specificity for CD44v6 (NCT03009214). In addition, a specific inhibitor of ALDH (NYH817G) is being tested as a monotherapy in patients with advanced solid tumors in which approved standard therapies have failed (NCT04262739). Moreover, other strategies, such as Antibody-Drug Conjugates (ADC), aim to achieve the specific targeting of CSCs [163]. In this context, different ADCs directed against LGR5 were developed and tested in preclinical studies in gastrointestinal tumors, with encouraging results being obtained [164,165]; however, they have not yet been tested in the clinical setting.

Regarding therapeutic strategies oriented to the targeting of cells expressing gCSC markers, it should be considered that, in general, these markers are also expressed in normal adult stem cells and even in subsets of differentiated cells within tissues. For instance, CD133 expression is rare in normal tissues but is expressed in some adult hematopoietic, neural and prostate SCs, whilst CD44 is found in most epithelial and lymphatic tissues as well as in hematopoietic, adipose and mesenchymal SCs [166]. The fact that this expression is not restricted to gCSCs could lead to marker-targeted therapies having side effects. Therefore, it is necessary to evaluate the tolerance to these therapies, but it is not ruled out that the non-fully selective targeting of gCSCs can lead to improvements in the prognosis of GC patients without causing severe side effects.

Furthermore, when applying therapies, an aspect that should not be ignored is the fact that treatments can promote the appearance of new CSCs. Radiotherapy and different chemotherapeutic agents, such as pemetrexed, cisplatin or doxorubicin, induce senescence in a fraction of tumor cells. This event is called therapy-induced senescence (TIS) and restrains tumor growth. It has been shown that senescent cells acquire the senescence- associated secretory phenotype (SASP) and secrete different factors (interleukins, chemokines, etc.) that can activate the immune response, promoting tumor clearance. However, SASP can induce the epithelial-to-mesenchymal transition and stemness in neighboring cells [167]. Furthermore, recent works have revealed that the non-stem tumor cells which release from senescence and re-enter the cell cycle, exhibit a stem cell expression pattern and increased tumor initiation capacity in vivo [168,169]. This senescence-associated reprogramming of cells has relevant implications for disease recurrence and suggests that it is worth assessing the use in the adjuvant setting of senolytic agents, which eradicate senescent cells. In this context, the most promising senolytic agent in preclinical studies is navitoclax, an inhibitor of members of the BcL-2 family, but there are some concerns and its implementation still requires further research [170].

In the therapeutic field, an attractive idea is to target circulating CSCs in order to prevent the establishment of metastases. In this regard, it has been shown that in GC the presence of detectable CTCs in the blood is an independent predictor of reduced survival [171,172]. Moreover, the CTC count after therapy predicts therapeutic response, recurrence and survival [173,174,175,176]. Particularly, CTCs expressing CD44 are the ones that predict GC prognosis and metastasis [101], which moreover, exhibit stem properties such as chemoresistance and multipotency ex vivo [89]. Since growing evidence indicates that CTC clusters present a much higher metastatic potential compared to single CTCs [177], a strategy oriented to disaggregate these clusters could be considered. In fact, some steps have already been taken in this regard in breast cancer [178], wherein a clinical trial evaluating the efficacy of the glycoside digoxin for the disruption of CTCs is currently in development (NTC03928210).

There are many strategies under study that could represent opportunities for gCSC targeting. The successful implementation of any of these strategies in combination with conventional current therapies, which are able to eliminate differentiated tumor cells, would represent the success in the effective eradication of tumors. In this context, it is true that gCSC targeting is still a challenging goal, but a deeper understanding of the regulation of these cells could make it possible.

## 5. Conclusions

gCSCs, due to their unique characteristics, are decisive in therapy failure. gCSCs exhibit an unlimited self-renewal capacity and are able to differentiate producing heterogeneous tumor cells. Therefore, this population of cells sustains tumors and represents an outstanding source of tumor heterogeneity. gCSCs exert diverse mechanisms that make them unsusceptible to conventional therapies, which are mainly directed against cells in proliferation. In line with this, their state of quiescence renders antimitotic drugs ineffective. Moreover, gCSCs exhibit the capacity to efflux a broad plethora of substances and drugs thanks to the high expression of membrane transporters. This fact limits the benefits of the chemotherapeutic agents since therapeutic concentrations are not reached in these cells. Furthermore, gCSCs develop different mechanisms that minimize drug-induced damages. In this regard, gCSCs present an enhanced DNA repair activity and are able to efficiently mitigate aldehydes and ROS. In addition, gCSCs survive upon injury due to molecular mechanisms that allow them to evade the induction of apoptosis. Therefore, there is an urgent need to identify agents that are effective against gCSCs. If therapies eliminate the differentiated tumor cells that conform the tumor bulk, but cannot eradicate gCSCs due to their resistance, tumor recurrence and dissemination phenomena and, ultimately, death, will occur.

Different gCSC regulators have been associated with therapy resistance and dismal prognosis in GC. These include LGR5, CD44, CD133, HMMR, E2F1 and ALDH. Diverse approaches aimed at targeting/inhibiting these regulators have been assessed, but so far none of these strategies or drugs have been approved for the treatment of cancer patients. Therefore, a deeper understanding is needed to elucidate how to make this population of cells drugable/targetable.

## Supplementary Materials

A glossary of the genes that are named in the work are available online at https://www.mdpi.com/article/10.3390/cancers14061457/s1, Table S1: Gene glossary.
