# Supplementary material for: Impact of Cancer Stem Cells on Therapy Resistance in Gastric Cancer
Source: Cancers (Basel). 2022 Mar 11;14(6):1457. doi: 10.3390/cancers14061457 (PMC8946717; doi:10.3390/cancers14061457)
Supplement: Supplementary file 1 [file cancers-14-01457-s001.zip › cancers-1610061-supplementary.pdf]

Table S1: Gene glossary.

Gene Symbol	Official Full Name	Gene ID
ALDH1A1 (ALDH1)	Aldehyde dehydrogenase 1 family member A1	216
ALDH1A3	Aldehyde dehydrogenase 1 family member A3	220
ALDH1L1	Aldehyde dehydrogenase 1 family member L1	10840
ALK	ALK receptor tyrosine kinase	238
AKT (AKT1)	AKT serine/threonine kinase 1	207
APC	APC regulator of WNT signaling pathway	324
ARID1A	AT-rich interaction domain 1A	8289
CCNE1	Cyclin E1	898
CD133 (PROM1)	Prominin 1	8842
CD166 (ALCAM)	Activated leukocyte cell adhesion molecule	214
CD44	CD44 molecule	960
CDH1	Cadherin 1	999
c-FLIP (CFLAR)	CASP8 and FADD like apoptosis regulator	8837
E2F1	E2F transcription factor 1	1869
EGFR	Epidermal growth factor receptor	1956
ERBB2 (HER2)	erb-b2 receptor tyrosine kinase 2	2064
ERCC1	ERCC excision repair 1, endonuclease non-catalytic subunit	2067
HMMR (CD168)	Hyaluronan mediated motility receptor	3161
JAK2	Janus kinase 2	3717
Klf4	Kruppel-like factor 4	16600
KRAS	KRAS proto-oncogene, GTPase	3845
LGR5	Leucine rich repeat containing G protein-coupled receptor 5	8549
MLH1	mutL homolog 1	4292
MTOR	Mechanistic target of rapamycin kinase	2475
MYC	MYC proto-oncogene, bHLH transcription factor	4609
PD-1 (PDCD1)	Programmed cell death 1	5133
PD-L1 (CD274)	CD274 molecule	29126
PD-L2 (PDCD1LG2)	Programmed cell death 1 ligand 2	80380
PIK3CA	Phosphatidylinositol-4,5-bisphosphate 3-kinase catalytic subunit alpha	5290
PLK1	Polo like kinase 1	5347
RHOA	RAS homolog family member A	387
RIF1	Replication timing regulatory factor 1	55183
RSK1 (RPS6KA1)	Ribosomal protein S6 kinase A1	6195
SMAD4	SMAD family member 4	4089
Sox2	SRY (sex determining region Y)-box 2	20674
TP53	Tumor protein p53	7157
UBE2 (UBA7)	Ubiquitin like modifier activating enzyme 7	7318
VEGFR2 (KDR)	Kinase insert domain receptor	3791
